# Neurology

**Published:** 1900-07

**Authors:** William C. Krauss

**Affiliations:** Buffalo, N. Y.


### NEUROLOGY.

Conducted by WILLIAM C. KRAUSS, M. D., Buffalo, N. Y.

### ADDICTION TO DRUGS, ESPECIALLY IN REFERENCE TO THE MEDICAL PROFESSION.

Dewey, Richard, of Wauwatosa, Wis. (*Medical Age*), states that the statistics as to morphine habitués treated in Prussian sanitariums show that of sixty-two male patients, almost one-third were physicians, and of eighteen female patients, three were wives of physicians.

My experience is that in twenty-eight cases of morphine and cocaine addiction under my care in four and a half years, thirteen were physicians, a percentage of forty-six. Taking physicians and the sporting fraternity together, the percentage would have been seventy.

The sporting class and demi-monde are specially prone to drugs as well as alcoholic excesses. I would emphasise, however, the fact that a great difference exists as to the reason for these excesses in one case and the other. The physician is wearing himself out in loyal and unselfish service; the other class in vicious and licentious courses. He is watching at the sick bed, while they are reveling; he takes something to brace himself for a new effort for humanity, while they are engaged in plundering and pandering.

The conclusion arrived at is that the percentage may be overestimated or underestimated, and is likely never to be accurately determined. There is, nevertheless, far more inebriety of this kind than appears on the surface. It is also likely to be overestimated in the public mind and an unjust suspicion to attach to the profession. As a professional body we should not only be virtuous, but above suspicion, and if necessary should promptly repudiate drug habits and those who practice them.

I have not sought to say anything here of the interesting subjects of diagnosis and symptoms of drug habits, of prognosis or of therapeutic means—all of which merit new and thorough study, and concerning which I hope to give my experience at another time.

I will close with a word concerning the most important subject of all—prophylaxis. It should be an inflexible rule with every physician neither to prescribe nor use the drugs that induce vicious habits except when absolutely indispensable and when absolutely controlled by himself alone, and, furthermore, never to employ these powerful agents of good or evil in his own person except when prescribed for him on each occasion by a brother physician in whose skill and integrity he may place absolute confidence.

---

### SCHOOL BREAK-DOWNS.

BARTLETT, J. HENRY, of Philadelphia, (*Medical News*, June 16, 1900,) presented a paper on this subject before the Section on Diseases of Children, A. M. A., Atlantic City, June 6, 1900.

Reference was made to an article in a first-class monthly in which the writer claimed that 16,000 children in five large cities had been compelled to leave school in one term because of breaking down in health. The belief that this result, as claimed by the writer, is due to the present school system is unwarranted. Causes other than school-life which play a large part in ill-health, causing the child to leave school were considered. The principal ones are diet and haste in eating, lack of exercise, and the hours of sleep. These all occurring during the developmental period cause deterioration in the child's condition. Whatever the school strain may do, it has a host of allies. In some cases the school may be a safety-valve where the evil effects of some of the other influences may be set aside. A thorough study of the developmental processes needs to be made to get at the bottom of what the writer of the article in question calls a crime. The school system is good in many ways; too many examinations, etc., are simply an overdoing of a principle and are not essentially a condemnation of the principle itself.

---

### ETIOLOGY AND TREATMENT OF CHOREA.

MONCORVO (*Pediatrics*) presents the following as to the etiology and treatment of chorea:

1. The frequency with which nervous and other symptoms are met in chorea places beyond doubt their influence as etiological factors.

2. Hysteria appears to exercise a marked influence on the onset of chorea, so much so that some authors have regarded the latter a form of it.

3. According to certain writers who have remarked the close connection between chorea and the infectious diseases common to a secondary affection, it is the sequel of those virulent diseases.

4. On one hand, the close relation between chorea and rheumatism, so long admitted by clinicians of the highest standing, and, on the other, the bacteriological nature of rheumatism as proved by Achalme, Thiroloix, Triboulet, Coyon and Ladoc, lead him to see in chorea only the cerebro-medullary tendency of a rheumatic infection developed in the hysterical or neurasthenic temperament.

5. Of the numerous remedies to which resort has been made in the treatment of chorea, the author gives the preference to those derived from the aromatic series, such as antipyrin (the proper dose of which he has determined), exalgine, asaprol and analgine.

6. While these remedies have been of undeniable efficiency in the treatment of rheumatism, it is equally true that under their action all choreic manifestations disappear within the space of from eighteen to fifty days.

7. The author's personal observations have assured him of the successful outcome of this treatment, at least in the case of children whose subsequent history he has been able to follow.

---

### THE NERVOUS SYSTEM IN PULMONARY PHTHISIS AS A BASIS FOR TREATMENT.

MAYS, THOMAS J., Philadelphia, (*Medical News*) read a paper with this title before the American Climatological Society, at Washington, May 3, 1900.

The author holds that phthisis is an exhaustion of the vital forces of the body in which loss of appetite, fever, nightsweats and other well-known symptoms accompany or precede the earliest implication of the pulmonary organs; he believes that the original trouble in the great majority of cases lies in the brain and nervous system and particularly in the pneumogastric nerves. That the vagi are primarily involved in phthisis Dr. Mays has shown in rather a large collection of cases in which confirmation was made post-mortem. Holding these views as to the etiology and pathology of phthisis, Dr. Mays discards all the usual remedies and employs those measures which stimulates the nervous system, such as strychnine, static electricity, quinine, capsicum, etc. He also employs massage of the vagi in the neck and subcutaneous injections along their course. The dose thus used was five minims of a 2½ per cent. solution of silver nitrate, preceded by a similar dose of a cocaine solution. The technic was described. Dr. Mays has used silver nitrate injections in about 200 cases of phthisis during the last nineteen months. The results were gratifying, the cough and expectoration were greatly relieved or ceased altogether, dyspnea and oppression of the chest were relieved; vomiting increased in a marked degree; the general strength improved. The effect on the physical signs was equally gratifying. This was particularly true of the gain in body weight. Dr. Mays believes that if this auxiliary method is combined with other good hygienic, dietetic and medicinal treatment the practical results will be greater than without that method, and he claims that the nervous system is the principal avenue through which pulmonary phthisis may be successfully approached from a therapeutic standpoint.

---

### ETIOLOGY OF PARESIS.

KRAFFT-EBING, VON R., of Vienna, (*American Journal of Insanity*, April, 1900,) in an excellent article says:

Enough has been said to prove beyond question that progressive paralysis, in the present day, is a scourge which decimates modern society. But, as a fact of no less importance, it is to be noted that the disease now seeks out its victims at a much earlier period of their lives. Calmeil, in France, in the twenties of the present century, found 44.5 years to be the average age for the onset of paresis. At the end of the eighties, Arnaud found it to be 39.5; Regis, exactly 38; whereas Kaes, in Hamburg, concluded that the majority of cases occurred between the ages of 36 and 40 years. This earlier onset of the disease has to be explained by assuming not only a greater prevalence of certain etiological factors, but also a lessened resistance to their baneful influences.

Still more startling is the occurrence of the disorder in childhood and youth, which has only lately been noted. As an illustration of this point, it may be said that whereas Baillarger before 1850 found among 400 female paretics only one under 20 years of age, in the literature since 1877 numerous instances of juvenile paresis are recorded.

Perhaps one of the most ominous features in this connection is to be found in the increase of paresis among females. Until well on in the sixties the number of male to female paretics in insane asylums stood in the proportion of 8 to 1; at the end of the seventies, according to Jung (Leubus), it was as 4 to 1. Early in the eighties it was calculated by Reinhard, for Hamburg, at 3.2 to 1; by Meynert, for Vienna, 3.4 to 1; by Siemerling, for Berlin, 3.5 to 1.

For the beginning of the nineties, Idanoff established it for various countries as follows: For Denmark, 3.9 to 1; for middle and upper Italy, 3.3 to 1; for Russia, 3.15 to 1; for England, 2.98 to 1; for Belgium, 2.76 to 1; for France, 2.4 to 1. These figures, however, certainly do not accurately represent the incidence of paresis among women at the present day, since the disorder occurs in them very frequently under the form of a simple dementia and often runs so mild a course that many of the cases are never confined in insane asylums and are consequently not included in the above-mentioned figures.

---

### PARETIC DEMENTIA.

THE following is a report (*American Journal of Insanity*, April, 1900,) of some of the features of forty-three cases of paretic dementia admitted in an eastern Michigan asylum, Pontiac, during the past three years :

Admitted January 1, 1897, to January 1, 1898, 22 cases—10 per cent. of all admissions.

Admitted January 1, 1898, to January 1, 1899, 15 cases—10 per cent. of all admissions.

Admitted January 1, 1899, to January 1, 1900, 6 cases—5 per cent. of all admissions.

Admitted January 1, 1897, to January 1, 1900, 43 cases—9 per cent. of all admissions.

Knee-jerk absent in 8 cases, 19 per cent.; present in 8 cases, 19 per cent; knee-jerk active in 27 cases, 64 per cent. Superficial reflexes absent in one case; present in 19 cases, 45 per cent; active in 8 cases, 36 per cent.

Causation: Syphilis in 27 cases, 62 per cent;; intemperance in 6 cases, 1 per cent; unknown 3 cases, 1 per cent; doubtful 4 (probably syphilitic), 1 per cent.

Pupils: Pupils regular, 9 cases, 22 per cent; irregular, 24 cases, 57 per cent.; right larger, 14 cases; left larger, 10 cases; Argyll-Robertson, 4 cases, 11 per cent.; bilateral dilatation, 1 case; no reaction of left pupil, 1 case; no reaction of right, 1 case; hippus, 2 cases; immobile, 10 cases, 28 per cent.

---

IF THE gall-bladder is so seriously diseased as to warrant surgical relief, it is better practice to remove it in toto than to leave it as a place for the further accumulation of gall-stones, or as a point for the setting up of new inflammations.—*International Journal of Surgery*.

